# On a New Method of Opening Abscesses, without Leaving Visible Cicatrices

**Published:** 1850-12

**Authors:** 

**Affiliations:** Physician to the Lyons Dispensary


### SURGERY.

*On a new Method of opening Abscesses, without leaving visible Cicatrices.* By M. LERICHE, Physician to the Lyons Dispensary.—The inconveniences daily met with in opening abscesses by incision, or by the application of caustics, have induced me to seek a less objectionable method of effecting this object. My principal aim has been to avoid the permanent marks left by the means hitherto employed, a point of much importance when the abscess occupies the neck or bosom of the female. I shall be much gratified if I can prove to the profession, what I am myself convinced of, that if my results have not been crowned with complete success, my patients have at least been often spared the dread which always attends the use of cutting instruments.

Although the method which I propose is apparently sufficiently simple, I have not arrived at it without repeated trials, both of the use of different materials and of the mode of operating. I shall, however, give in a few words the result of my researches.

My first idea was to employ wires of iron, silver, or lead; the results were tolerably satisfactory, but their use was liable to three objections:

1st. The difficulty of procuring them everywhere.

2nd. The necessity of having a special instrument for their introduction, and the rather acute pain which the operation occasioned.

3rd, and lastly, the contact of a hard substance, irritating the inflamed and already painful tissues.

I also tried threads of hemp, linen, and cotton; all were liable to a serious objection, which induced me to discard them altogether; they became swollen by the moisture in which they were constantly immersed, and thus opposed the exit of the pus. I remarked also that their employment gave rise to a rather acute inflammation around the openings. Might not this be attributed to the facility with which these substances become altered in their nature? It also occurred when the threads were previously waxed.

Silk thread is the material on which I have decided, from its having the following advantages over the others. 1st. It is to be had everywhere. 2ndly. It is not liable to become altered during the time it is required to remain in the abscess. 3rdly. It does not absorb the moisture. 4thly, and lastly, it does not irritate the painful parts with which it is in contact. The silk thread which I use is known in the shops under the name of twist (cordonnet).

After having shaved off any hair on the tumor, the surgeon takes a curved ligature needle, passes through its eye one end of the silk twist, then introduces the needle into the tumour, about six or eight lines from the most depending part, where it must be brought out, draws the thread into the passage formed by the needle, and retains it in this position by uniting the two ends in a knot; the entire tumour is now covered with an emollient poultice, which, in this case, acts mechanically. The patient should remain as little as possible in bed, in order to favour the escape of the pus along the thread, an effect which takes place with difficulty in the recumbent position, when the abscess is situated on a part of the trunk or limbs. The poultices have also, in this case, the advantage of diminishing the inflammation which is excited, and which the practitioner must watch. The twist is to be left undisturbed for four, six, or eight days, according to circumstances; most frequently four days have sufficed. Subsequently, when thought advisable, the twist is removed, and the part is dressed with dry compresses, or, when necessary, with compresses soaked in aromatic wine. In not one of thirty-three buboes which had arrived at the stage of suppuration, and which had been treated in the manner just described, have I been obliged to abandon this plan for any other. In cases of simple buboes, that is, those in which the pus did not seem to possess specific characters, the cure has been effected in from fifteen to twenty days; in the opposite cases, when the orifices of the little openings ulcerated, it has occupied from forty to fifty days; and in neither case did the patient retain any trace of syphilitic infection.

When the tumour has been tardy in reaching the suppurative stage, the pus is sometimes contained in cellular pouches, in which case it may happen that at the time when one thread seems to have effected the cure of an abscess, another forms; under these circumstances a second thread must be introduced.

In other cases, the thread has brought on severe inflammation and caused intense pain. When this occurs it must be removed, and lightly astringent unctuous applications, such as Goulard's cerate, substituted for the poultices; to these should be added the employment of general measures, baths, regimen, &c. &c.

But the selection of the means most suitable to combat the symptoms which may have arisen must, in such cases, be left to the judgement of the practitioner.—*Dub. Quar. Journal, from Révue Medico-Chirurgicale de Paris.*

